# FBXW11 contributes to stem-cell-like features and liver metastasis through regulating HIC1-mediated SIRT1 transcription in colorectal cancer

**DOI:** 10.1038/s41419-021-04185-7

**Published:** 2021-10-12

**Authors:** Jing Yao, Jun Yang, Zhe Yang, Xin-Ping Wang, Tong Yang, Bing Ji, Zheng-Yun Zhang

**Affiliations:** 1 Department of Surgery, Shanghai Jiao Tong University Affiliated Sixth People’s Hospital, Shanghai City, 200233 China; 2 Department of Internal Medicine, Huzhou Traditional Chinese Medicine Hospital Affiliated to Zhejiang Chinese Medical University, Huzhou City, Zhejiang Province 313000 China

**Keywords:** Cancer, Endocrine system and metabolic diseases

## Abstract

Colorectal tumorigenesis is a heterogeneous disease driven by multiple genetic and epigenetic alterations. F-box and WD repeat domain containing 11 (FBXW11) is a member of the F-box protein family that regulates the ubiquitination of key factors associated with tumor growth and aggressiveness. Our study aimed to explore the role of FBXW11 in the development and metastasis of colorectal cancer (CRC). FBXW11 was overexpressed in colorectal tumor tissues and its overexpression was associated with a poor prognosis of CRC patients. The upregulation of FBXW11 not only promoted cell proliferation, invasion, and migration, but also contributed to maintaining stem-cell features in colorectal tumor cells. Further analysis revealed that FBXW11 targeted hypermethylated in cancer 1 (HIC1) and reduced its stability in CRC cells through ubiquitination. Moreover, the expression of sirtuin 1 (SIRT1), a deacetylase in tumor cells was upregulated by FBXW11 via regulating HIC1 expression. The mouse xenograft models of CRC confirmed that FBXW11 knockdown impeded colorectal tumor growth and liver metastasis in vivo. In summary, our study identified FBXW11 as an oncogenic factor that contributed to stem-cell-like properties and liver metastasis in CRC via regulating HIC1-mediated SIRT1 expression. These results provide a rationale for the development of FBXW11-targeting drugs for CRC patients.

## Introduction

Colorectal cancer (CRC) is a lethal malignancy worldwide with nearly 900,000 deaths per year [1]. The age-standardized incidence of CRC is 19.7 per 100,000 people, making it the third most common carcinoma in both men and women [2]. Due to the growing aging population and increasing dietary fat intake in recent decades, the prevalence of CRC has been elevating rapidly in Asian countries, including China [3]. Colorectal carcinogenesis is driven by multiple genetic and epigenetic alterations [4]. Intra-tumor heterogeneity is a hallmark of CRC and has been considered a major problem limiting the efficacy of current therapies [5]. Cancer stem cells (CSCs) represent a subgroup of cells within a tumor that are phenotypically and functionally heterogeneous and highly dynamic [6]. The self-renewal and asymmetrical differentiation capacities of CSCs are essential for tumorigenesis, metastasis, and treatment resistance [7]. Therefore, therapeutic strategies targeting stem-cell-like features in tumor cells may present a promising approach for CRC patients.

F-box and WD repeat domain containing 11 (FBXW11) is a substrate adaptor of an E3 ubiquitin ligase complex that catalyzes phosphorylation-dependent ubiquitination and proteasomal degradation in tumorigenesis-related signaling pathways [8]. Previous evidence showed that FBXW11 accelerated lymphocytic leukemia cell growth through concomitant activation of the β-catenin/T-cell factor and the nuclear factor-κB (NF-κB) signaling pathways [9]. Bhatia et al. [10] reported the upregulation of FBXW11 in skin tumors and found that FBXW11 promoted skin carcinogenesis by targeting the ubiquitination of inhibitor of NF-κB (IκB), thereby activating the NF-κB signaling. FBXW11 is also implicated in the proliferation and migration/invasion of cervical cancer cells as a target of microRNA [11]. In contrast, the suppression of FBXW11 and FBXW7 in non-small cell lung cancer cells has been shown to promote tumor growth [12]. Major et al. [13] reported that WTX, a protein encoded by a gene mutated in Wilms tumors, forms a complex with FBXW11, AXIN1, β-catenin, and adenomatous polyposis coli (APC), which promoted β-catenin ubiquitination and thus antagonizes the WNT/β-catenin signaling, in cultured human colon carcinoma cells, *Xenopus*, and zebrafish. Zhu et al. [14] reported that FBXW11 interacted with Zinc finger protein 281 (ZNF281) and promoted its degradation, thereby inhibiting CRC growth and metastasis [14]. However, they only examined a small number of CRC tissue samples using semi-quantitative method and only focused on the effect of FBXW11 on ZNF281-mediated CRC cell growth. Hence, more comprehensive understanding of the role of FBXW11 in colorectal carcinogenesis is needed.

*Hypermethylated in cancer 1* (HIC1) is a tumor suppressor gene located telomeric to the *p53* gene at chromosome 17p13.3 [15]. The epigenetic silencing of the *HIC1* gene has been frequently observed in different types of human malignancies, including gastric and liver cancers, esophageal cancers, and colon cancers [16]. Sirtuin 1 (SIRT1) is a NAD-dependent deacetylase that can form a complex with HIC1, which binds to the *SIRT1* promoter, represses its transcription, thereby regulating p53-dependent apoptotic DNA damage [17]. A study by Cheng et al. [18] showed that SIRT1 promoted epithelial–mesenchymal transition and metastasis in CRC via upregulating transcription factor Fra-1. The aforementioned results highlighted the function of SIRT1 as a therapeutic target for CRC treatments.

The present study investigated the regulatory effects of FBXW11 on stem-cell-like features and liver metastasis in CRC. The involvement of HIC1 and SIRT1 during FBXW11-mediated colorectal tumor growth was also explored. Our results provide a rationale for exploring the clinical use of FBXW11-targeting drugs for CRC patients.

## Materials and methods

### Ethical statement

The use of clinical samples was approved by the Ethics Committee of the Shanghai Jiao Tong University Affiliated Sixth People’s Hospital. The experimental procedures were performed according to the Declaration of Helsinki [19]. All participants provided written informed consent before enrollment. The experimental procedures regarding animal handling were approved by the Animal Care and Use Committee of Shanghai Jiao Tong University Affiliated Sixth People’s Hospital and were performed in accordance with the Guide for the Care and Use of Laboratory Animals [20].

### Clinical samples

One hundred and forty-five pairs of colorectal tumor samples and adjacent non-tumorous tissues were obtained from CRC patients who underwent surgical tumor resection. There were 76 males and 69 females in this cohort, with an average age of 66.7 ± 4.2 years. Tissue specimens were prepared for subsequent immunohistochemistry analysis, quantitative real-time PCR (qRT-PCR), and western blotting. CRC patients were further divided into two groups according to the mRNA or protein level of FBXW11 in tumor tissues. Patients with FBXW11 level below the median value of all tested individuals were assigned to the FBXW11-low group, whereas those with FBXW11 level above the median value were categorized as the FBXW11-high group. The disease-free and overall survival were calculated by the Kaplan–Meier method and compared using the log-rank test.

Tissue samples subjected to immunohistochemistry analysis were embedded in paraffin, cut into 5 µm sections, and incubated with the primary antibodies (Abcam, Cambridge, USA) at 4 °C overnight: anti-FBXW11 antibody (1 : 50; catalog number 137835), anti-aldehyde dehydrogenase 1 (ALDH1) antibody (1 : 50; catalog number 52492), and anti-SIRT1 antibody (5 µg/mL; catalog number 110304). The next day, tissue sections were stained with a secondary antibody (1 : 2000; catalog number 6721, Abcam) for half an hour at room temperature. The immunostaining intensity and the percentage of positively stained cells were counted by two experienced pathologists who were blinded to the clinical data and experimental design. A 0–3 scale was used to determine the immunohistochemical intensity score as follows: 0, no staining; 1, slightly yellow (weak) staining; 2, brown–yellow (moderate) staining; and 3, brown (strong) staining. The percentage of positively stained cells was counted and graded as follows: 0, no staining; 1, <10% cells exhibiting positive staining; 2, 10–35% cells were positively stained; 3, 35–70% positively stained cells; and 4, >70% cells exhibiting positive staining. The staining index (SI) was calculated by multiplying the immunohistochemical intensity score and the positive staining score. An SI of ≤4 was considered low expression, whereas an SI of ≥6 was defined as high expression.

### CRC cell culture and transfection

Human colon mucosal epithelial cells NCM460 and different human colorectal tumor cell lines (LoVo, HTC116, SW480, T84, SW620) were purchased from ATCC (Manassas, USA) and cultured in a 5% CO_2_/37 °C incubator. NCM460, HCT116, and T84 were maintained in McCoy’s 5A medium (Invitrogen, Carlsbad, USA), whereas LoVo, SW480, and SW620 cells were cultured in RPMI-1640 medium (Invitrogen) containing 10% fetal bovine serum (FBS). Recombinant lentiviral vectors carrying FBXW11 or HIC1, and corresponding control vectors (named Control and mock, respectively) were designed and generated by GeneChem (Shanghai, China). Short hairpin RNAs (shRNAs) targeting FBXW11 (shFBXW11-1 and shFBXW11-2), SIRT1 (shSIRT1), and HIC1 (shHIC1), as well as corresponding scrambled controls (named Scramble and shRNA, respectively) were synthesized by GenePharma (Shanghai, China). Lipofectamine® 2000 Transfection Reagent (Invitrogen) was used to transfect colorectal tumor cell cells with designated lentiviral vectors and/or shRNAs. Transfection efficiency was evaluated by qRT-PCR and/or western blotting prior to further analyses.

### Mouse models of colorectal tumor growth and liver metastasis

A mouse CRC model was established using male BALB/c nude mice (Charles River Laboratories, Wilmington, USA) at 6 weeks old. All animals were housed in a controlled environment (12 h light–dark cycle, 50% humidity, 23 ± 2 °C) with free access to water and food. After 1-week acclimation, mice were assigned into four groups (*n* = 6/group). HCT116 and SW620 cells were transfected with shRNA targeting FBXW11 or scrambled control sequence as mentioned above. Transfected cells (5 × 10^6^) were then collected, washed with phosphate-buffered saline (PBS), resuspended in 200 μL PBS mixed with Matrigel (9 : 1, v/v), and injected subcutaneously into the flank of the target mouse. Tumor volume was calculated as follows:

$${{{\mathrm{Tumor}}}}\;{{{\mathrm{volume}}}}\,\left( {{{{\mathrm{mm}}}}^3} \right) = (\pi \times {{{\mathrm{length}}}} \times {{{\mathrm{width}}}}^2)/6$$

and recorded every 5 days for 35 days. On the last day, all animals were killed and tumor xenografts were collected for immunohistochemical staining. Tumor specimens were embedded in paraffin, cut into 5 µm sections, and stained with FBXW11 (1 : 50; catalog number 137835, Abcam), SIRT1 (5 µg/mL; catalog number 110304, Abcam), HIC1 (20 µg/mL; catalog number 271499, Santa Cruz Biotechnology, Dallas, USA), and Ki-67 (5 µg/mL; catalog number 15580, Abcam). The stained tumor tissue sections were examined under a light microscope.

A splenic liver metastasis model was established by injecting male BALB/c nude mice (8-week-old, Charles River Laboratories) with colorectal tumor cells into the spleen as previously described [21]. Briefly, mice (*n* = 6 per group) were anesthetized with 2% isoflurane and then a lateral incision was made to expose the spleen. Following transfection with shRNA targeting FBXW11 (or scrambled control sequence), SW620 cells (2 × 10^5^) were collected, resuspended in 10 μL PBS, and injected into the spleen using a 33-gauge micro-injector (Hamilton Company, Reno, USA). The injection site was sealed with 3 M Vetbond tissue adhesive (3 M Animal Products, St. Paul, USA). The abdominal wall and the skin were closed with a 6-0 polyglycolic acid suture (Accutome, Inc., Malvern, USA). All animals were killed 3 weeks after implantation. Livers were collected, weighed, and processed for hematoxylin and eosin staining (Sigma-Aldrich, St. Louis, USA).

### Assessment of cell viability and colony formation ability

Cell viability was determined by MTT (3-(4,5-dimethylthiazol-2-yl)-2,5-diphenyltetrazolium bromide) assay (Roche, Mannheim, Germany). HCT116 and SW620 cells were plated in 96-well plates (5 × 10^3^ cells/well) and transfected with designated lentiviral vectors and/or shRNAs as aforementioned. At 0, 24, 48, 72, and 96 h post transfection, 10 μL of 0.5 mg/mL MTT solution was added to cells and incubated for 3 h. Next, 100 μL of solubilization buffer was added to cells and the absorbance at 490 nm was measured by a microplate reader (R&D Systems, Minneapolis, USA). Colony formation assay was performed to evaluate the colony formation ability of colorectal tumor cells following transfection. Transfected HCT116 and SW620 cells were seeded in six-well plates (5 × 10^2^ cells/well) and maintained in normal culture medium supplemented with 10% FBS for 2 weeks. Then, cells were fixed with 4% paraformaldehyde and stained with 0.2% crystal violet (Sigma-Aldrich). The number of colonies per well was counted.

### Wound-healing assay and Transwell assay

The migration capacity of colorectal tumor cells was determined by wound-healing assay. HCT116 and SW620 cells were plated in six-well plates and transfected with designated lentiviral vectors and/or shRNAs when reached ~70% confluence. Forty-eight hours later, a straight scratch was made using a sterile 20 μL pipette tip. Cells were then maintained in serum-free medium for 24 h. The wound width at 24 h after scratching relative to that at 0 h was measured. In Transwell invasion assay, transwell insert was coated with 50 µL Matrigel (Corning, Inc., Corning, USA) for 20 min at 37 °C. Then 100 μL of cell suspension containing ~1 × 10^6^ cells were added to the top of the coating. The upper chamber of the plate was filled with 600 μL serum-free medium, whereas the bottom chamber was added with the same volume of medium containing 10% FBS. Transwell insert was removed from the plate at 24 h post treatment, washed with PBS to remove residual medium and cells, and then stained with 2% crystal violet. The number of invaded cells was counted from six randomly selected fields.

### Sphere formation assay

The sphere-forming ability of colorectal tumor cells was evaluated by sphere formation assay as previously reported [22]. Briefly, one single tumor cell was sorted into one well of the Corning® Costar® ultra-low attachment 96-well plate (Sigma-Aldrich), which was filled with 200 μL of Dulbecco’s modified Eagle medium/F12 medium supplemented with 20 μg/mL fibroblast growth factor-basic, 1× B27, and 20 μg/mL epidermal growth factor (Life Technologies, Waltham, USA). Cells were cultivated in a 5% CO_2_/37 °C for 7 days. The number of spheres with a diameter of >50 μm was counted and normalized to the Control group.

### Cell sorting and flow cytometry

Transfected HCT116 and SW620 cells (~1 × 10^7^) were collected using non-enzymatic cell dissociation solution (Sigma-Aldrich), resuspended in 1 mL staining buffer (Miltenyi Biotec, Bergisch Gladbach, Germany), and then incubated with mouse anti-human CD44-FITC antibody and mouse anti-human CD133-APC antibody (Miltenyi Biotec) for 10 min. Then, stained cells were sorted by flow cytometry using a BD FACSMelody^TM^ Cell Sorter (BD Biosciences, San Jose, USA). The percentage of cells expressing surface markers CD133 and CD44 were calculated.

To detect the apoptotic rate of CRC cells following transfection, cells were collected, centrifuged, and resuspended by 500 μL 1× binding buffer. Then, cells were incubated with 5 μL V-FITC and 5 μL propidium iodide (Biovision Research Products, Mountain View, USA) for 5 min in the dark. The apoptotic rate was then analyzed by a flow cytometer (Beckman Coulter, Brea, USA).

### Co-immunoprecipitation

HEK-293T cells (1 × 10^6^) were cultured in a 15 cm Petri dish to reach ~70% confluence and then transfected with FLAG-tagged FBXW11 or HA-tagged HIC1, or both for 48 h [23]. Subsequently, transfected cells were lysed with 2 mL lysis buffer (Cell Signaling Technology, Danvers, USA), centrifuged at 13,000 r.p.m. for 10 min, and incubated with 35 μL of 50% suspension containing antibodies against FLAG tag or HA tag (Sigma-Aldrich) at 4 °C for 3 h. Then, 40 μL of sample buffer was added to immunoprecipitates and immunoprecipitated proteins were analyzed by western blotting.

### Ubiquitination and stability of HIC1

The ubiquitination level of HIC1 in colorectal tumor cells with varied FBXW11 expression was examined by treating them with MG132 (20 μM; Sigma-Aldrich). Dynabeads^TM^ Magnetic Beads (Thermo Fisher Scientific) were conjugated with anti-HIC1 antibody (catalog number 271499, Santa Cruz Biotechnology) for 15 min. After 3 h of incubation with MG132, colorectal tumor cells were lysed and mixed with the conjugated beads for 30 min. Subsequently, the immunoprecipitates were separated by 10% SDS-polyacrylamide gel electrophoresis (SDS-PAGE) and stained with anti-poly-ubiquitin antibody (catalog number 7780, Abcam). The cycloheximide (CHX) chase assay was performed to assess the stability of HIC1 in colorectal tumor cells following transfection. HCT116 and SW620 cells were transfected with lentiviral vectors or shRNAs for 48 h. The 10 µg/mL CHX (Sigma-Aldrich) was added to cells, to inhibit de novo protein synthesis in transfected tumor cells. The protein level of Sox9 was measured by western blotting at 0–12 h after treatment.

### Quantitative real-time PCR

Total RNA was extracted from human tissue samples and cell lysates using TRIzol extraction kit (Invitrogen). ReverTra Ace qPCR RT kit (Toyobo, Osaka, Japan) was used for cDNA synthesis. qRT-PCR was performed on a 7300 RT-PCR System (Applied Biosystem, Foster City, USA). The sequences of the primers were as follows: FBXW11 forward, 5′-CCGACTCGGTGATTGAGGAC-3′, FBXW11 reverse, 5′-CCGATGTTCCCCACATCCAA-3′; HIC1 forward, 5′-CCCGGGACTGATAATGTGA-3′, HIC1 reverse, 5′-AGACCTGGTGGTAGGCTCTT-3′; SIRT1 forward, 5′-GGTTGACTTAGGTCTTGTCTG-3′, SIRT1 reverse, 5′-CGTCCCTTGTAATGTTTCCC-3′; GAPDH (internal control) forward: 5′-ATCACTGCCACCCAGAAGAC-3′ GAPDH reverse: 5′-TTTCTAGACGGCAGGTCAGG-3′.

### Western blotting

Total protein was isolated from human tissue samples and colorectal tumor cells using lysis buffer containing protease inhibitors (Pierce Biotechnology, Waltham, USA). Equal amounts of protein samples were separated on a 10% SDS-PAGE gel and then blotted with the following primary antibodies (Abcam unless indicated otherwise): FBXW11 (1 : 500; catalog number 137835), ALDH1 (1 : 500; catalog number 52492), Nanog (1 : 1000; catalog number 109250), Oct4 (1 : 1000; catalog number 181557), HIC1 (1 : 500; catalog number 271499, Santa Cruz Biotechnology), SIRT1 (1 µg/mL; catalog number 110304), and β-actin (internal control; 1 : 2000, catalog number 32572). After 1 h incubation with a secondary antibody (1 : 2000; catalog number 6721, Abcam), the blots were visualized by the Alphalmager^TM^ 2000 Imaging System (Alpha Innotech, San Leandro, USA). The band density was quantified using ImageJ.

### Statistical analysis

All experiments were performed three times, each performed in triplicate. Data were analyzed by the SPSS software (version 24.0) and presented as mean ± SD. Pearson’s correlation coefficient was used to analyze the linear relationship between continuous variables. Student’s *t*-test and one-way analysis of variance followed by Bonferroni’s post hoc test were used for statistical comparisons between two groups or among multiple groups, respectively. A *p*-value < 0.05 indicated statistical significance (*p < 0.05, **p < 0.01, ***p < 0.01).

## Results

### FBXW11 is upregulated in CRC tissues

**Fig. 1:**
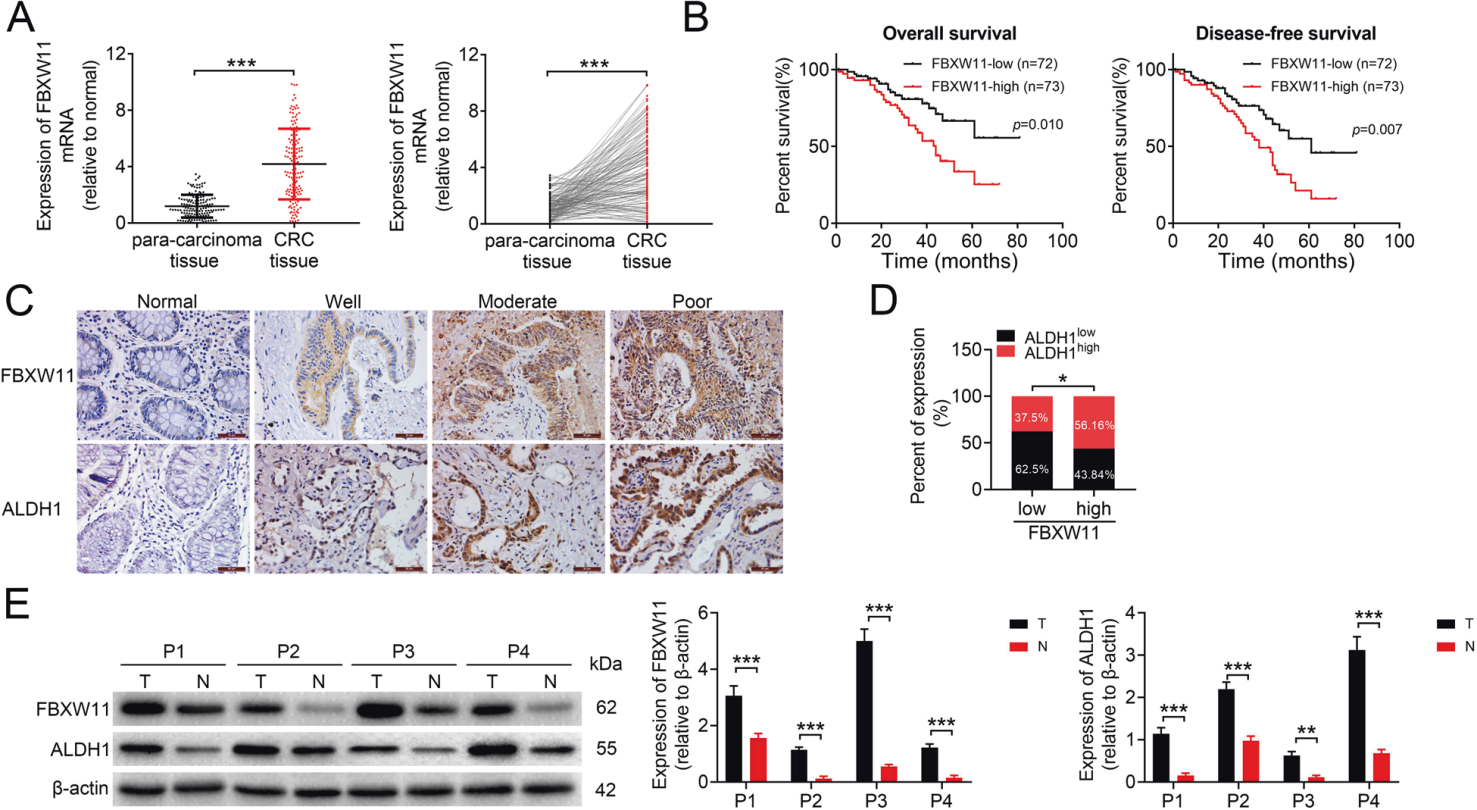
Expression of FBXW11 in CRC tissue samples and its correlation with the prognosis of CRC patients. Colorectal tumor tissue and paired adjacent non-tumorous tissue samples were collected from 145 patients with CRC. **A** The mRNA level of FBXW11 in tissue specimens was measured by qRT-PCR. **B** The overall and disease-free survival of CRC patients with low or high FBXW11 expression was calculated using the Kaplan–Meier method. **C** The expressions of FBXW11 and ALDH1 in colorectal and adjacent non-tumorous tissue samples were detected by immunohistochemistry. Tissue samples with no staining (Normal, adjacent tissues), weak staining (Well), moderate staining (Moderate), and strong staining (Poor) were shown (×400 magnification). **D** The SI of each section was calculated by multiplying the immunohistochemical intensity score and the score of positively stained cells. An SI of ≤4 was defined as low expression, whereas an SI of ≥6 was considered high expression. The box plot shows the percentage of samples with low or high ALDH1 expression in tumors with low or high FBXW11 level. **E** Representative blots show the protein expressions of FBXW11 and ALDH1 in four pairs of tissue samples. Student’s *t*-test was used for statistical comparisons between the two groups.

To explore whether FBXW11 was involved in the progression of CRC, we first analyzed the mRNA expression of FBXW11 in 145 pairs of colorectal tumor tissues and paired non-tumorous tissues. The result showed that FBXW11 was significantly upregulated in colorectal tumors (Fig. 1A). Then, CRC patients were divided into two groups (FBXW11-low and FBXW11-high) according to the medium value of FBXW11 level in all tested tumor samples. Patients with low FBXW11 expression exhibited significantly better overall survival and disease-free survival compared to those with FBXW11 expression above the median value (Fig. 1B). The protein level of FBXW11 in clinical tissue samples was also measured and the overall survival according to the FBXW11 protein expression was consistent with that based on the mRNA level (Supplementary Fig. 1A). Immunohistochemistry analysis revealed that FBXW11 and ALDH1 (a stem cell marker) were abundantly expressed in colorectal tumor tissues but not in adjacent normal tissues (Fig. 1C). By analyzing the SI of tumor tissue sections stained with FBXW11 and ALDH1, we found that tumors with high FBXW11 expression were more likely to overexpress ALDH1 and vice versa (Fig. 1D). Western blot analysis confirmed that both FBXW11 and ALDH1 were upregulated in colorectal tumor tissues compared to paired non-tumorous tissues (Fig. 1E). These findings imply that FBXW11 may exert an oncogenic effect in the development of CRC.

### FBXW11 promotes CRC cell growth and migration

**Fig. 2:**
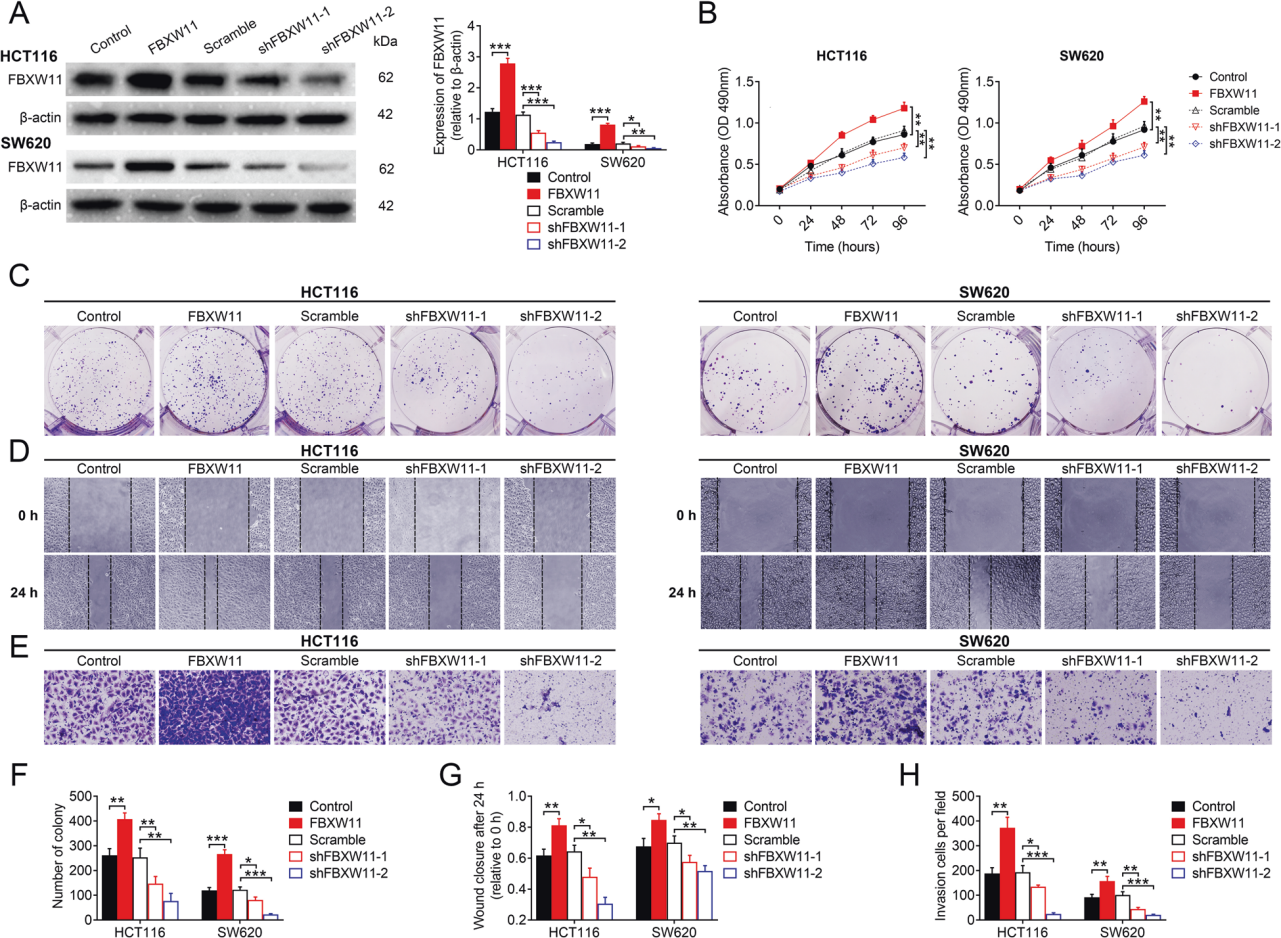
Effect of FBXW11 on the growth and migration of CRC cells. Human CRC cell lines HCT116 and SW620 were transfected with recombinant lentiviral vectors carrying FBXW11 or shRNAs targeting FBXW11 (shFBXW11-1 and shFBXW11-2). The control groups were transfected with corresponding empty control vectors or scrambled shRNA sequence. **A** Transfection efficiency was evaluated by western blotting at 48 h post transfection. **B** The MTT assay was used to evaluate cell viability at 0, 24, 48, 72, and 96 h after transfection. **C**, **F** The colony formation assay was performed by culturing transfected CRC cells with culture medium supplemented with 10% FBS for 2 weeks. The number of colonies per well was counted. **D**, **G** The wound-healing assay was performed to evaluate the migration capacity of transfected CRC cells. The wound closure at 24 h post scratching was measured. **E**, **H** The Transwell assay was used to assess the invasion capacity of CRC cells. Cells were seeded in Matrigel-coated chambers filled with serum-free medium. The bottom chamber was added with the same volume of normal culture medium. After 24 h, the number of invaded cells per group was counted from six randomly selected fields (×400 magnification). ANOVA followed by Bonferroni’s post hoc test was used for statistical comparisons among multiple groups.

To investigate the effects of FBXW11 expression on colorectal tumor growth, we first measured the level of FBXW11 in different CRC cells compared with normal colon mucosal epithelial cells NCM460. We found that FBXW11 was highly expressed in all CRC cell lines. The expression of FBXW11 in SW620 and HCT116 cells was neither the highest nor the lowest; therefore, they were suitable for further overexpression and knockdown experiments (Supplementary Fig. 1B). Then, we transfected CRC cell lines HCT116 and SW620 with vectors carrying FBXW11 (or control vectors) or shRNAs targeting FBXW11 (or scrambled control sequence). Cells delivered with FBXW11-carrying vectors exhibited significantly higher expression of the target gene, whereas those transfected with shRNAs exhibited markedly decreased FBXW11 expression compared with the corresponding controls (Fig. 2A). The upregulation of FBXW11 significantly increased the viability (Fig. 2B), promoted the colony formation capacity (Fig. 2C, F), and inhibited the apoptosis (Supplementary Fig. 1C) of colorectal tumor cells, whereas FBXW11 deficiency suppressed cell growth and proliferation. In both CRC cell lines, FBXW11 overexpression effectively enhanced cell migration (Fig. 2D, G) and invasion (Fig. 2E, H), whereas the downregulation of FBXW11 significantly impaired the motility of tumor cells. These data indicate that FBXW11 is an indispensable prerequisite for colorectal tumor cell growth and migration.

### FBXW11 facilitates the maintenance of stem-cell-like properties in CRC cells

**Fig. 3:**
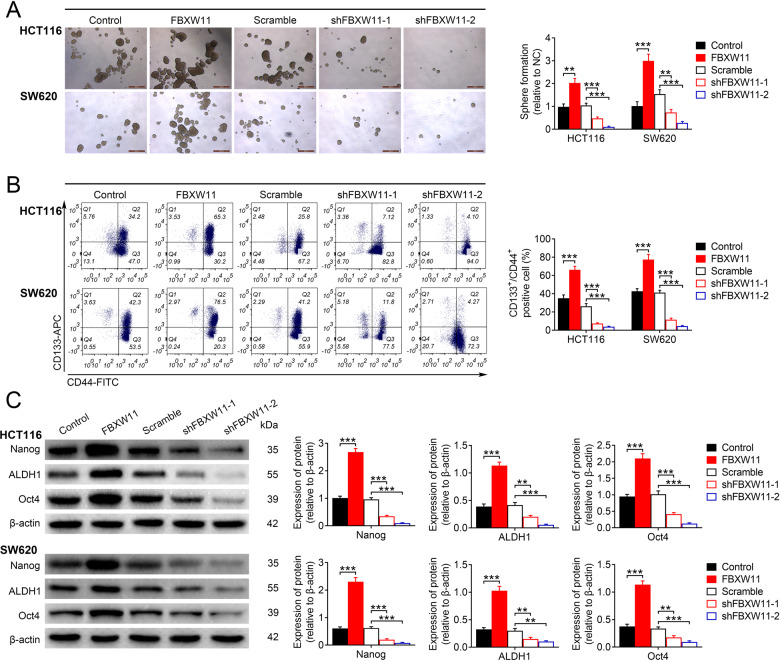
Effect of FBXW11 on maintaining stem-cell-like properties in CRC cells. HCT116 and SW620 cells were transfected with recombinant lentiviral vectors carrying FBXW11, shRNAs targeting FBXW11, or corresponding controls. **A** The sphere-forming ability of CRC cells following transfection was evaluated by cultivating one single cell in growth medium for 7 days. The number of spheres (diameter >50 μm) were counted and normalized to the group transfected with empty control lentiviral vector. **B** Transfected CRC cells were stained with anti-CD44-FITC and anti-CD133-APC antibodies, and then sorted by flow cytometry. The percentage of CD133^+^/CD44^+^ cells in each group was calculated. **C** The protein expressions of Nanog, ALDH1, and Oct4 were measured by western blotting. ANOVA followed by Bonferroni’s post hoc test was used for statistical comparisons among multiple groups.

We further explored whether FBXW11 would affect the maintenance of stem-cell-like features in colorectal tumor cells. Compared to cells with normal FBXW11 expression, those transfected with vectors carrying FBXW11 showed significantly enhanced sphere-forming ability. The downregulation of FBXW11, however, greatly suppressed sphere formation in both HCT116 and SW620 cells (Fig. 3A). Furthermore, the percentage of cells expressing CSC markers (i.e., CD44 and CD133) in the group transfected with FBXW11-carrying vectors was significantly higher than that in the empty vector-transfected group. However, the group with insufficient FBXW11 expression had significantly fewer CD133^+^/CD44^+^ cells in comparison to the controls (Fig. 3B). In addition, the protein expression of stem cell markers, including Nanog, ALDH1, and Oct4 in different groups, was measured by western blotting. All these markers were highly upregulated in FBXW11-overexpressing cells, but dramatically inhibited in the group with insufficient FBXW11 expression (Fig. 3C). The above results imply that FBXW11 is essential for the maintenance of stem-cell-like properties in CRC cells.

### FBXW11 reduces the stability of HIC1

**Fig. 4:**
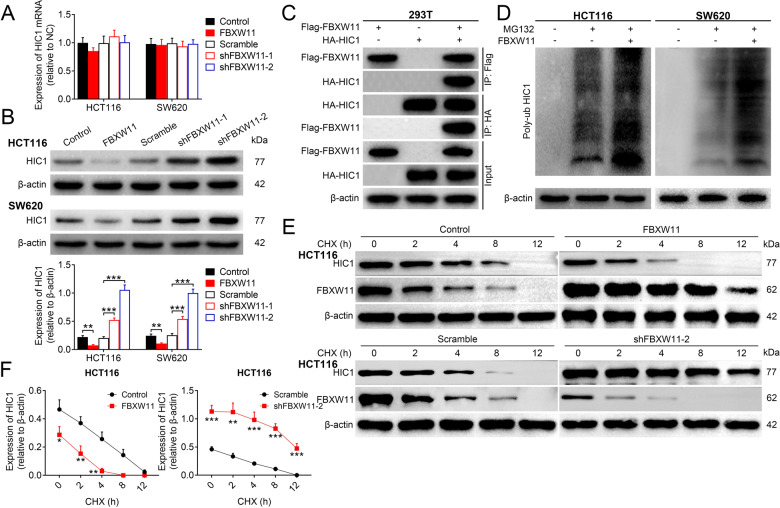
Effect of FBXW11 on the stability of HIC1. **A**, **B** HCT116 and SW620 cells were transfected with recombinant lentiviral vectors carrying FBXW11, shRNAs targeting FBXW11, or corresponding controls. The **A** mRNA and **B** protein levels of HIC1 in transfected CRC cells were determined by qRT-PCR and western blotting, respectively. **C** HEK-293T cells were transfected with FLAG-tagged FBXW11 or HA-tagged HIC1, or both for 48 h. Then cells were stained antibodies against FLAG tag or HA tag. The immunoprecipitated proteins were analyzed by western blotting. **D** The ubiquitination level of HIC1 in transfected CRC cells was examined by treating them with 20 μM of MG132 for 3 h. Cells were then mixed with conjugated beads followed by immunoprecipitation with anti-poly-ubiquitin antibody. **E** Transfected HCT116 and SW620 cells were treated with 10 µg/mL of CHX. The protein expression of HIC1 was analyzed by western blotting at 0, 2, 4, 8, and 12 h after treatment. **F** The qualification curves of HIC1 expression in both cells lines at different time points were plotted. Student’s *t*-test and ANOVA followed by Bonferroni’s post hoc test were used for statistical comparisons between two groups or among multiple groups, respectively.

Next, we examined whether HIC1 was implicated in FBXW11-mediated colorectal tumor growth. The qRT-PCR analysis revealed that there was no significant difference in the mRNA level of HIC1 among groups with varied FBXW11 expression (Fig. 4A). However, the protein expression of HIC1 was markedly decreased in cells overexpressing FBXW11, but markedly elevated in the group with FBXW11 deficiency (Fig. 4B), suggesting that the FBXW11 level might be correlated with the protein expression of HIC1 in CRC cells. The co-immunoprecipitation assay showed that the HIC1 protein was detectable in immunoprecipitates obtained by the anti-FLAG antibody. Meanwhile, the samples pulled down by the anti-HA antibody expressed the FBXW11 protein (Fig. 4C). Then, we detected the expression of HIC1 in CRC cells transfected with FBXW11-carrying vectors or empty vectors following MG132 treatment. We found that the addition of MG132 significantly increased the protein expression of HIC1 in both CRC cells, but this effect was completely abolished by the upregulation of FBXW11 (Supplementary Fig. 2). Subsequently, the effect of FBXW11 on the ubiquitination level of HIC1 in colorectal tumor cells was examined. The results demonstrated that FBXW11 promoted the ubiquitination level of HIC1 (Fig. 4D). To analyze the effect of FBXW11 on the stability of HIC1 in CRC cells, CHX chase assay was performed. FBXW11 overexpression accelerated the degradation of the HIC1 protein in tumor cells, whereas the knockdown of FBXW11 decelerated this process compared to cells with normal FBXW11 expression (Fig. 4E, F). Collectively, it could be concluded that FBXW11 reduces the stability of the HIC1 protein through ubiquitination.

### FBXW11 upregulates SIRT1 via suppressing the expression of HIC1

**Fig. 5:**
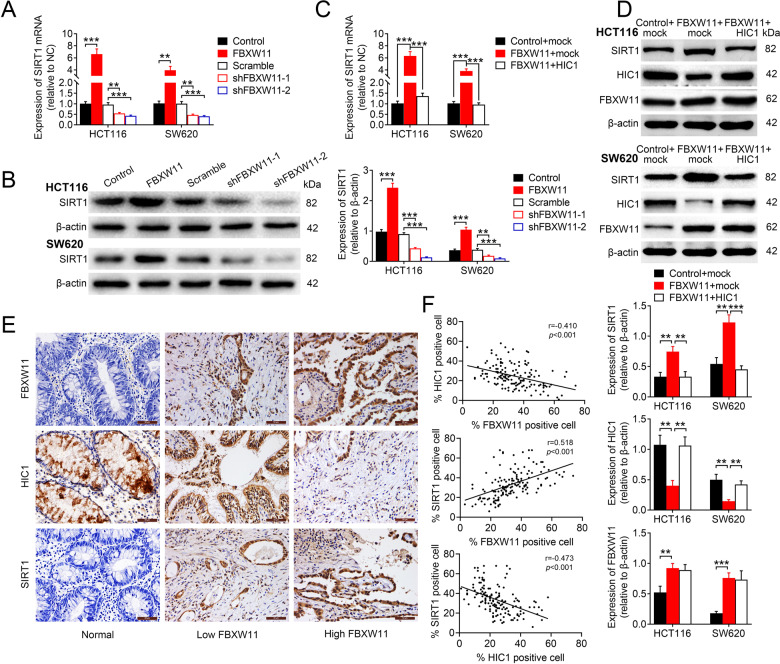
FBXW11 mediates the expression of HIC1 via SIRT1. **A**, **B** HCT116 and SW620 cells were transfected with recombinant lentiviral vectors carrying FBXW11, shRNAs targeting FBXW11, or corresponding controls. The **A** mRNA and **B** protein expressions of SIRT1 in transfected CRC cells were determined by qRT-PCR and western blotting, respectively. **C**, **D** HCT116 and SW620 cells were co-transfected with recombinant lentiviral vectors carrying FBXW11 (or empty control vectors) and vectors carrying HIC1 (or empty control vectors). The **C** mRNA and **D** protein expressions of SIRT1 were measured by qRT-PCR and western blotting, respectively. The protein expressions of HIC1 and FBXW11 were also detected. **E** The expressions of FBXW11, HIC1, and SIRT1 in human colorectal tumor tissue and adjacent non-tumorous tissue specimens were detected by immunohistochemistry (×400 magnification). Each set of slides (each column) was from the same subject. **F** The percentages of cells positively stained with FBXW11, HIC1, or SIRT1 were calculated. Their relationships were analyzed by Pearson’s correlation coefficient. ANOVA followed by Bonferroni’s post hoc test was used for statistical comparisons among multiple groups.

To further elucidate the mechanisms underlying the regulation of CRC progression by FBXW11, we measured the expressions of SIRT1 in HCT116 and SW620 cells overexpressing or repressing FBXW11. SIRT1 was significantly upregulated in cells overexpressing FBXW11, but downregulated in the group transfected with shRNAs targeting FBXW11 (Fig. 5A, B). Moreover, the delivery of HIC1-carrying vectors into CRC cells with sufficient FBXW11 expression significantly inhibited SIRT1 expression at both mRNA (Fig. 5C) and protein (Fig. 5D) levels. The transfection efficiency was confirmed by measuring the expression levels of FBXW11 and HIC1 (Fig. 5D). The immunohistochemistry staining revealed that the expressions of FBXW11 and SIRT1 in human colorectal tumors were positively correlated (Fig. 5E). In addition, the level of HIC1 was negatively correlated with the expressions of FBXW11 and SIRT1 (Fig. 5F). In addition, the knockdown of HIC1 in both HCT116 and SW620 cell lines with insufficient FBXW11 expression significantly elevated the expression level of SIRT1, further supporting the HIC1-dependent regulation of SIRT1 (Supplementary Fig. 1D). These results suggest that FBXW11 may upregulate the expression of SIRT1 in CRC cells through HIC1.

### FBXW11 maintains stem-cell-like properties of CRC cells via SIRT1

**Fig. 6:**
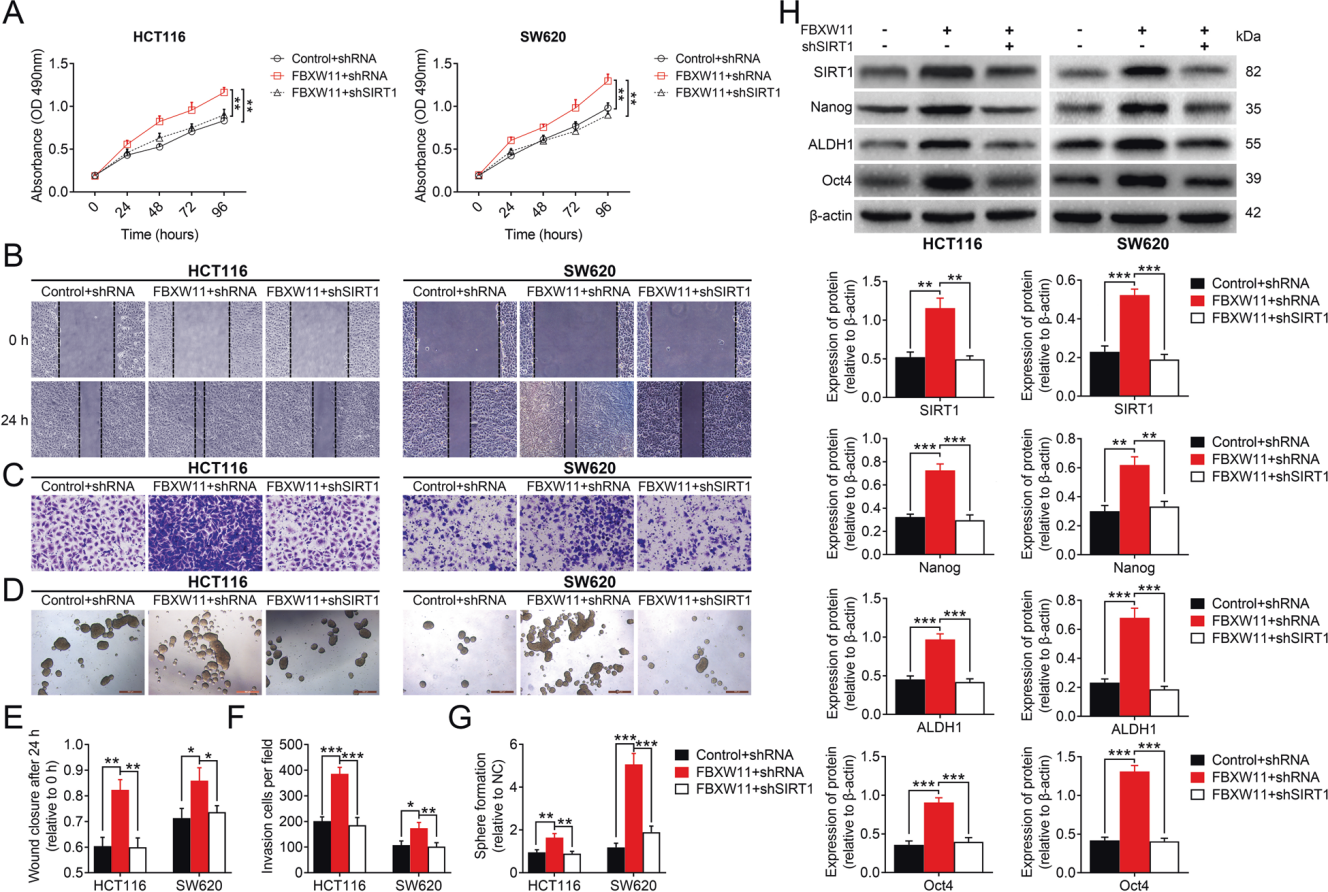
FBXW11 mediates stem-cell-like properties of CRC cells via SIRT1. HCT116 and SW620 cells were co-transfected with recombinant lentiviral vectors carrying FBXW11 (or empty control vectors) and shRNA targeting SIRT1 (or scrambled shRNA sequence). **A** Cell viability at different time points after transfection was evaluated by the MTT assay. **B**, **E** The wound-healing assay was used to assess the migration capacity of CRC cells following transfection. The wound closure at 24 h after scratching was measured. **C**, **F** The invasion capacity of transfected CEC cells was determined by the Transwell assay. The number of invaded cells per field at 24 h post plating was calculated. **D**, **G** The sphere-forming ability of CRC cells following transfection was evaluated by the sphere formation assay. The number of spheres with a diameter of over 50 μm was counted. **H** The protein expressions of Nanog, ALDH1, and Oct4 were assessed by western blotting. ANOVA followed by Bonferroni’s post hoc test was used for statistical comparisons among multiple groups.

To ascertain the involvement of SIRT1 in FBXW11-mediated colorectal tumor growth, we co-transfected CRC cells with vectors carrying FBXW11 (or control vectors) and shRNA targeting SIRT1 (or scrambled shRNA sequence). The downregulation of SIRT1 significantly reduced the viability of tumor cells overexpressing FBXW11 (Fig. 6A). The enhanced migration (Fig. 6B, E) and invasion (Fig. 6C, F) capacities of FBXW11-overexpressed cells were completely impeded by the knockdown of SIRT1. The upregulation of FBXW11 significantly promoted sphere formation of both HCT116 and SW620 cells, whereas the delivery of shRNAs targeting SIRT1 notably inhibited the sphere-forming ability of these cells (Fig. 6D, G). The measurement of CSC markers demonstrated that the downregulation of SIRT1 effectively inhibited FBXW11-induced upregulation of these proteins (Fig. 6H). However, the knockdown of SIRT1 did not alter the expressions of FBXW11 or HIC1 in these cells (Supplementary Fig. 1E). Taken together, FBXW11 promotes cell growth and migration, maintains stem-cell-like properties in CRC cells via regulating SIRT1.

### FBXW11 knockdown inhibits colorectal tumor growth and liver metastasis

**Fig. 7:**
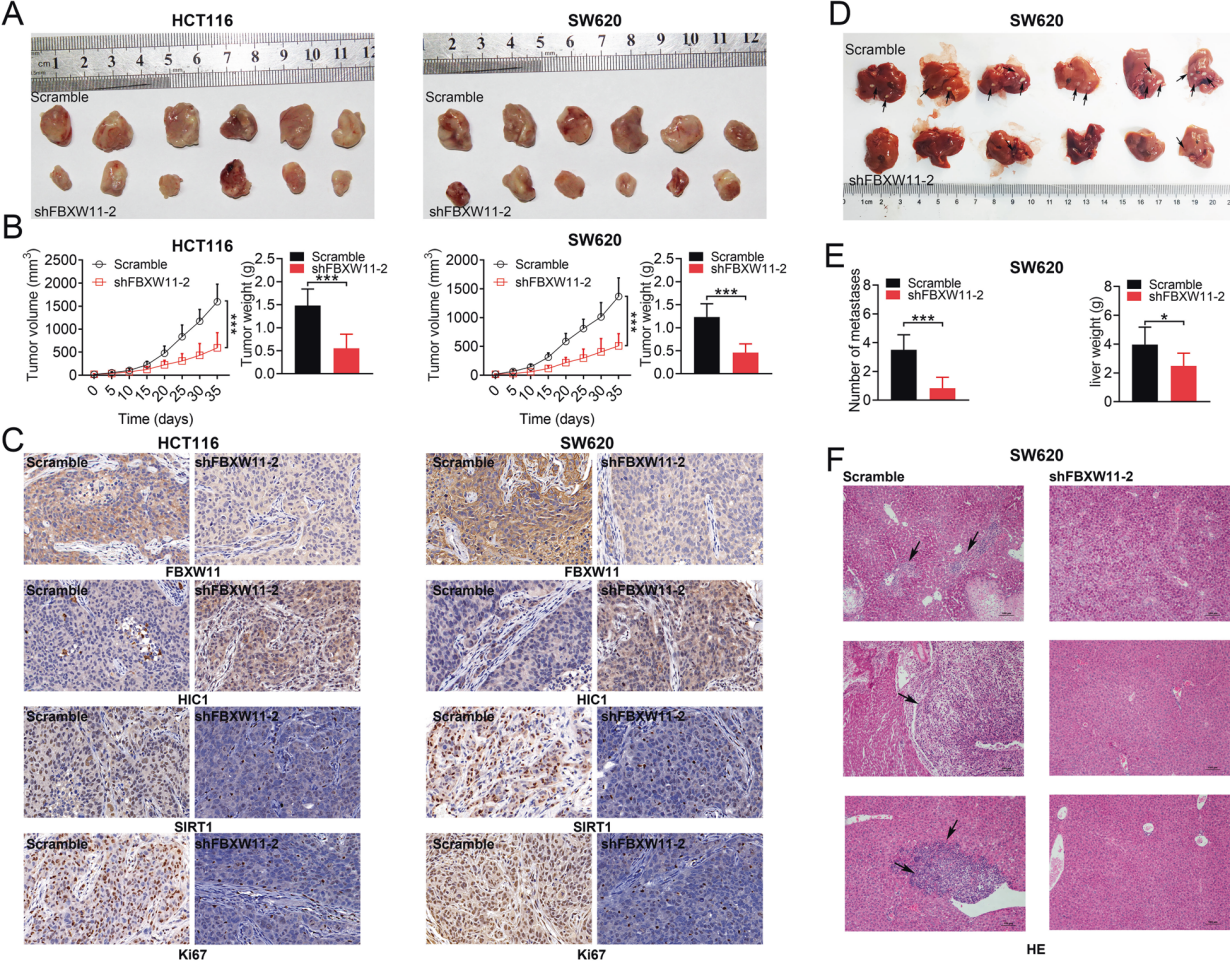
Effect of FBXW11 knockdown on colorectal tumor growth and liver metastasis. **A**–**C** A mouse CRC xenograft model was established by injecting male BALB/c nude mice with transfected CRC cells into the subcutaneous region of the flank (*n* = 6 per group). Tumor growth was monitored for 35 days. **A** Tumor xenografts were collected and weighed at the end of the experiment. **B** Tumor volume was recorded every 5 days and a growth curve was plotted. **C** Tumor tissues were sectioned and immunostained FBXW11, SIRT1, HIC1, and Ki-67 (200× magnification). **D**–**F** A splenic liver metastasis model was established by injecting male BALB/c nude mice with transfected CRC cells into the spleen (*n* = 6 per group). At 3 weeks post implantation, mice were killed and livers were collected. **D** Black arrows indicate metastatic foci in the liver. **E** The number of metastases was counted and the liver was weighed. **F** Liver tissues were sectioned and stained for H&E. Black arrows indicate metastases. Student’s *t*-test was used for statistical comparisons between two groups.

Lastly, the effect of FBXW11 on the development of CRC in vivo was investigated. A xenograft model was constructed by injecting BALB/c mice with HCT116 or SW620 cells with normal or insufficient FBXW11 expression. The group inoculated with FBXW11-deficient cells exhibited significantly reduced tumor volume and weight compared with the controls (Fig. 7A, B), suggesting that FBXW11 knockdown suppressed colorectal tumor growth in mouse xenografts. We further stained tumor samples with FBXW11, SIRT1, HIC1, and a proliferation marker, Ki-67. Mice implanted with shFBXW11-transfected cells exhibited much weaker staining of FBXW11, SIRT1, and Ki-67, but stronger staining of HIC1 compared to their counterparts in the control group (Fig. 7C). To evaluate the regulatory potential of FBXW11 on the metastasis of CRC, we established a splenic liver metastasis model by injecting BALB/c nude mice with transfected CRC cells into the spleen. After 3 weeks of implantation, the group inoculated shFBXW11-transfected cells showed significantly fewer metastases and lower liver weight compared to the control animals (Fig. 7D, E). The histological examination also confirmed that FBXW11 deficiency inhibited liver metastasis of CRC cells (Fig. 7F). The above findings indicate that FBXW11 knockdown not only suppresses colorectal tumor growth but also inhibits liver metastasis in vivo.

## Discussion

Similar to other malignancies, liver metastasis is the major cause of cancer-related mortality in CRC, which occurs in ~50% of all cases [24]. Although the long-term survival of CRC patients at early stages has been significantly improved in the past few years, the prognosis of patients with metastatic CRC remains poor, with an average 5-year survival below 15% [25]. In this study, we identified FBXW11 as an oncogenic factor that contributed to stem-cell-like properties and liver metastasis in CRC via regulating HIC1-mediated SIRT1 expression. These results highlighted the function of FBXW11 as a therapeutic target for CRC therapy.

FBXW11 plays a pivotal role in cancer development by governing the turnover of key proteins associated with tumor cell proliferation and mobility [26]. Elevated expression of FBXW11 has become a common trend in various human cancers, such as hematopoietic malignancy, prostate carcinoma, and breast cancer [27]. In the present study, FBXW11 was highly expressed in colorectal tumor tissues and its overexpression was associated with a poor prognosis of CRC patients. Previous studies have shown that FBXW11 exerts an oncogenic role by promoting tumor cell growth and migration (e.g., in leukemia and cervical cancer) [9, 11]. Similarly, we found that the upregulation of FBXW11 increased cell viability and promoted invasion and migration of colorectal tumor cells, whereas FBXW11 depletion suppressed cell proliferation and mobility. The xenograft models further confirmed that the downregulation of FBXW11 impeded colorectal tumor growth and inhibited liver metastasis in vivo.

Colorectal CSCs not only possess self-renewal and multi-directional differentiation potential but also demonstrate persistent activation of proliferation-related signaling pathways (i.e., Wnt, Hedgehog, Notch, etc.) and strong drug resistance, all of which contribute to CRC metastasis and relapse [28]. CD44 and CD133 are specific markers of primary colorectal CSCs associated with tumor growth, aggressiveness, and resistance in CRC [29, 30]. ALDH1 also serves as a stem cell marker in colorectal tumors, which is highly correlated with lymph node stage, tumor stage, and tumor differentiation [31]. Here we found that FBXW11 knockdown significantly inhibited the sphere-forming ability and the proportion of CD133^+^/CD44^+^ cells in CRC. Further analysis of ALDH1 and universal CSC markers (Nanog and Oct4) showed that all these markers were upregulated in CRC cells overexpressing FBXW11, but markedly downregulated in cells with insufficient FBXW11 expression. These results suggested a fundamental role of FBXW11 in maintaining stem-cell features in CRC cells.

Similar to other F-box proteins, FBXW11 regulates a multitude of cellular processes via selectively binding target proteins for ubiquitination. FBXW11 and its paralog FBXW1 have been shown to specifically interact with and promote the ubiquitination of Lipin1, a multifunctional phosphatidate phosphatase, in hepatocellular carcinoma cells, thereby promoting hepatic lipogenesis [32]. FBXW11 also targets key factors in transcriptional signaling pathways, such as β-catenin, GLI, and IκB, for ubiquitination and proteasomal degradation [33, 34]. In this study, we showed for the first time that FBXW11 targeted HIC1 and reduced its stability through ubiquitination.

*HIC1* is a tumor suppressor gene silenced in various human cancers, including CRC, in association with frequent DNA hypermethylation [35]. The epigenetic silence of *HIC1* through promoter hypermethylation maintain or promote a malignant phenotype in colon tumors in a lineage-specific manner [16, 36]. Previous evidence suggests that HIC1 hypermethylation may increase along with tumor progression and the level of hypermethylated HIC1 may correspond to tumor aggressiveness and poor prognosis [37]. SIRT1, as an important transcriptional target of HIC1, is transcriptionally upregulated in tumor cells due to the loss of HIC1 [38]. Sun et al. [39] reported that hypoxia repressed the transcription of SIRT1 in lung cancer cells by stimulating the binding of HIC1 to the *SIRT1* promoter. In our study, by measuring the SIRT1 expression in CRC cells co-transfected with vectors carrying FBXW11 and HIC1, and analyzing the correlations of FBXW11, HIC1, and SIRT1 expressions in colorectal tumors, we deduced that FBXW11 upregulated the expression of SIRT1 in CRC cells via reducing the stability of HIC1. A study by Lee et al. [40] indicated that SIRT1 was a fundamental contributor to oncogenic transformation of neural stem cells in glial tumors. Moreover, SIRT1 overexpression is significantly correlated with advanced-stage, lymph node or liver metastases, and poor prognosis in CRC patients [41]. Our data revealed that the downregulation of SIRT1 in CRC cells significantly suppressed FBXW11 overexpression-induced cell proliferation, motility, sphere formation, and upregulation of stem cell markers, implying that FBXW11 promoted CRC progression via SIRT1.

In summary, FBXW11 plays an indispensable role in promoting tumor growth, maintaining stem-cell-like features in tumor cells and inducing liver metastasis in CRC. The mechanism of action involves promoting ubiquitination-dependent degradation of HIC1, which results in SIRT1 upregulation in CRC cells. Therapeutic strategies targeting the FBXW11-HIC1-SIRT1 axis may be developed to delay or inhibit the metastasis of CRC.

## Supplementary information

supplemental material

## Data Availability

All data generated or analyzed during this study are included in this published article.

## References

[1] Araghi M, Soerjomataram I, Jenkins M, Brierley J, Morris E, Bray F (2019). Global trends in colorectal cancer mortality: projections to the year 2035. Int J Cancer.

[2] Rawla P, Sunkara T, Barsouk A (2019). Epidemiology of colorectal cancer: incidence, mortality, survival, and risk factors. Prz Gastroenterol.

[3] Xu R, Wang W, Zhu B, Lin X, Ma D, Zhu L (2020). Disease characteristics and treatment patterns of Chinese patients with metastatic colorectal cancer: a retrospective study using medical records from China. BMC Cancer.

[4] Grady WM, Markowitz SD (2015). The molecular pathogenesis of colorectal cancer and its potential application to colorectal cancer screening. Digest Dis Sci.

[5] Testa U, Pelosi E, Castelli G (2018). Colorectal cancer: genetic abnormalities, tumor progression, tumor heterogeneity, clonal evolution and tumor-initiating cells. Med Sci.

[6] Hirata A, Hatano Y, Niwa M, Hara A, Tomita H (2019). Heterogeneity of colon cancer stem cells. Adv Exp Med Biol.

[7] Prager BC, Xie Q, Bao S, Rich JN (2019). Cancer stem cells: the architects of the tumor ecosystem. Cell Stem Cell.

[8] Kim TY, Siesser PF, Rossman KL, Goldfarb D, Mackinnon K, Yan F (2015). Substrate trapping proteomics reveals targets of the βTrCP2/FBXW11 ubiquitin ligase. Mol Cell Biol.

[9] Wang L, Feng W, Yang X, Yang F, Wang R, Ren Q (2018). Fbxw11 promotes the proliferation of lymphocytic leukemia cells through the concomitant activation of NF-κB and β-catenin/TCF signaling pathways. Cell Death Dis.

[10] Bhatia N, Herter JR, Slaga TJ, Fuchs SY, Spiegelman VS (2002). Mouse homologue of HOS (mHOS) is overexpressed in skin tumors and implicated in constitutive activation of NF-kappaB. Oncogene.

[11] Zhang Q, Zheng J, Liu L (2019). The long noncoding RNA PCGEM1 promotes cell proliferation, migration and invasion via targeting the miR-182/FBXW11 axis in cervical cancer. Cancer Cell Int.

[12] Chang H, Liu YH, Wang LL, Wang J, Zhao ZH, Qu JF (2018). MiR-182 promotes cell proliferation by suppressing FBXW7 and FBXW11 in non-small cell lung cancer. Am J Transl Res.

[13] Major MB, Camp ND, Berndt JD, Yi X, Goldenberg SJ, Hubbert C (2007). Wilms tumor suppressor WTX negatively regulates WNT/beta-catenin signaling. Science.

[14] Zhu Y, Zhou Q, Zhu G, Xing Y, Li S, Ren N (2017). GSK-3β phosphorylation-dependent degradation of ZNF281 by β-TrCP2 suppresses colorectal cancer progression. Oncotarget.

[15] Wales MM, Biel MA, el Deiry W, Nelkin BD, Issa JP, Cavenee WK (1995). p53 activates expression of HIC-1, a new candidate tumour suppressor gene on 17p13.3. Nat Med.

[16] Fleuriel C, Touka M, Boulay G, Guérardel C, Rood BR, Leprince D (2009). HIC1 (Hypermethylated in Cancer 1) epigenetic silencing in tumors. Int J Biochem Cell Biol.

[17] Chen WY, Wang DH, Yen RC, Luo J, Gu W, Baylin SB (2005). Tumor suppressor HIC1 directly regulates SIRT1 to modulate p53-dependent DNA-damage responses. Cell.

[18] Cheng F, Su L, Yao C, Liu L, Shen J, Liu C (2016). SIRT1 promotes epithelial-mesenchymal transition and metastasis in colorectal cancer by regulating Fra-1 expression. Cancer Lett.

[19] World Medical Association Declaration of Helsinki. (2013). ethical principles for medical research involving human subjects. JAMA.

[20] National Research Council Committee for the Update of the Guide for the Care & Use of Laboratory Animals. Guide for the Care and Use of Laboratory Animals. 8th ed. Washington: National Academies Press; 2011.

[21] Zhang Y, Davis C, Shah S, Hughes D, Ryan JC, Altomare D (2017). IL-33 promotes growth and liver metastasis of colorectal cancer in mice by remodeling the tumor microenvironment and inducing angiogenesis. Mol Carcinogenesis.

[22] Fan F, Samuel S, Evans KW, Lu J, Xia L, Zhou Y (2012). Overexpression of snail induces epithelial-mesenchymal transition and a cancer stem cell-like phenotype in human colorectal cancer cells. Cancer Med.

[23] Zoncu R, Bar-Peled L, Efeyan A, Wang S, Sancak Y, Sabatini DM (2011). mTORC1 senses lysosomal amino acids through an inside-out mechanism that requires the vacuolar H(+)-ATPase. Science.

[24] Vatandoust S, Price TJ, Karapetis CS (2015). Colorectal cancer: metastases to a single organ. World J Gastroenterol.

[25] Brouwer NPM, Bos A, Lemmens V, Tanis PJ, Hugen N, Nagtegaal ID (2018). An overview of 25 years of incidence, treatment and outcome of colorectal cancer patients. Int J Cancer.

[26] Wang Z, Liu P, Inuzuka H, Wei W (2014). Roles of F-box proteins in cancer. Nat Rev Cancer.

[27] Fuchs SY, Spiegelman VS, Kumar KG (2004). The many faces of beta-TrCP E3 ubiquitin ligases: reflections in the magic mirror of cancer. Oncogene.

[28] Zhou Y, Xia L, Wang H, Oyang L, Su M, Liu Q (2018). Cancer stem cells in progression of colorectal cancer. Oncotarget.

[29] Kazama S, Kishikawa J, Kiyomatsu T, Kawai K, Nozawa H, Ishihara S (2018). Expression of the stem cell marker CD133 is related to tumor development in colorectal carcinogenesis. Asian J Surg.

[30] Lee SY, Kim KA, Kim CH, Kim YJ, Lee JH, Kim HR (2017). CD44-shRNA recombinant adenovirus inhibits cell proliferation, invasion, and migration, and promotes apoptosis in HCT116 colon cancer cells. Int J Oncol.

[31] Chen J, Xia Q, Jiang B, Chang W, Yuan W, Ma Z (2015). Prognostic value of cancer stem cell marker ALDH1 expression in colorectal cancer: a systematic review and meta-analysis. PLoS ONE.

[32] Shimizu K, Fukushima H, Ogura K, Lien EC, Nihira NT, Zhang J (2017). The SCFβ-TRCP E3 ubiquitin ligase complex targets Lipin1 for ubiquitination and degradation to promote hepatic lipogenesis. Sci Signal.

[33] Holt RJ, Young RM, Crespo B, Ceroni F, Curry CJ, Bellacchio E (2019). De novo missense variants in FBXW11 cause diverse developmental phenotypes including brain, eye, and digit anomalies. Am J Hum Genet.

[34] Shirane M, Hatakeyama S, Hattori K, Nakayama K, Nakayama K (1999). Common pathway for the ubiquitination of IkappaBalpha, IkappaBbeta, and IkappaBepsilon mediated by the F-box protein FWD1. J Biol Chem.

[35] Zheng J, Xiong D, Sun X, Wang J, Hao M, Ding T (2012). Signification of hypermethylated in cancer 1 (HIC1) as tumor suppressor gene in tumor progression. Cancer Microenviron.

[36] Szczepny A, Carey K, McKenzie L, Jayasekara WSN, Rossello F, Gonzalez-Rajal A (2018). The tumor suppressor Hic1 maintains chromosomal stability independent of Tp53. Oncogene.

[37] Markowski J, Sieroń AL, Kasperczyk K, Ciupińska-Kajor M, Auguściak-Duma A, Likus W (2015). Expression of the tumor suppressor gene hypermethylated in cancer 1 in laryngeal carcinoma. Oncol Lett.

[38] Lin Z, Fang D (2013). The roles of SIRT1 in cancer. Genes Cancer.

[39] Sun L, Li H, Chen J, Dehennaut V, Zhao Y, Yang Y (2013). A SUMOylation-dependent pathway regulates SIRT1 transcription and lung cancer metastasis. J Natl Cancer Inst.

[40] Lee JS, Park JR, Kwon OS, Lee TH, Nakano I, Miyoshi H (2015). SIRT1 is required for oncogenic transformation of neural stem cells and for the survival of “cancer cells with neural stemness” in a p53-dependent manner. Neuro Oncol.

[41] Lv L, Shen Z, Zhang J, Zhang H, Dong J, Yan Y (2014). Clinicopathological significance of SIRT1 expression in colorectal adenocarcinoma. Med Oncol.

