# Supplementary material for: FBXW11 contributes to stem-cell-like features and liver metastasis through regulating HIC1-mediated SIRT1 transcription in colorectal cancer
Source: Cell Death Dis. 2021 Oct 12;12(10):930. doi: 10.1038/s41419-021-04185-7 (PMC8511012; doi:10.1038/s41419-021-04185-7)
Supplement: Supplementary file 1 — supplemental material [file 41419_2021_4185_MOESM1_ESM.docx]

***Supplementary Figure 1.***


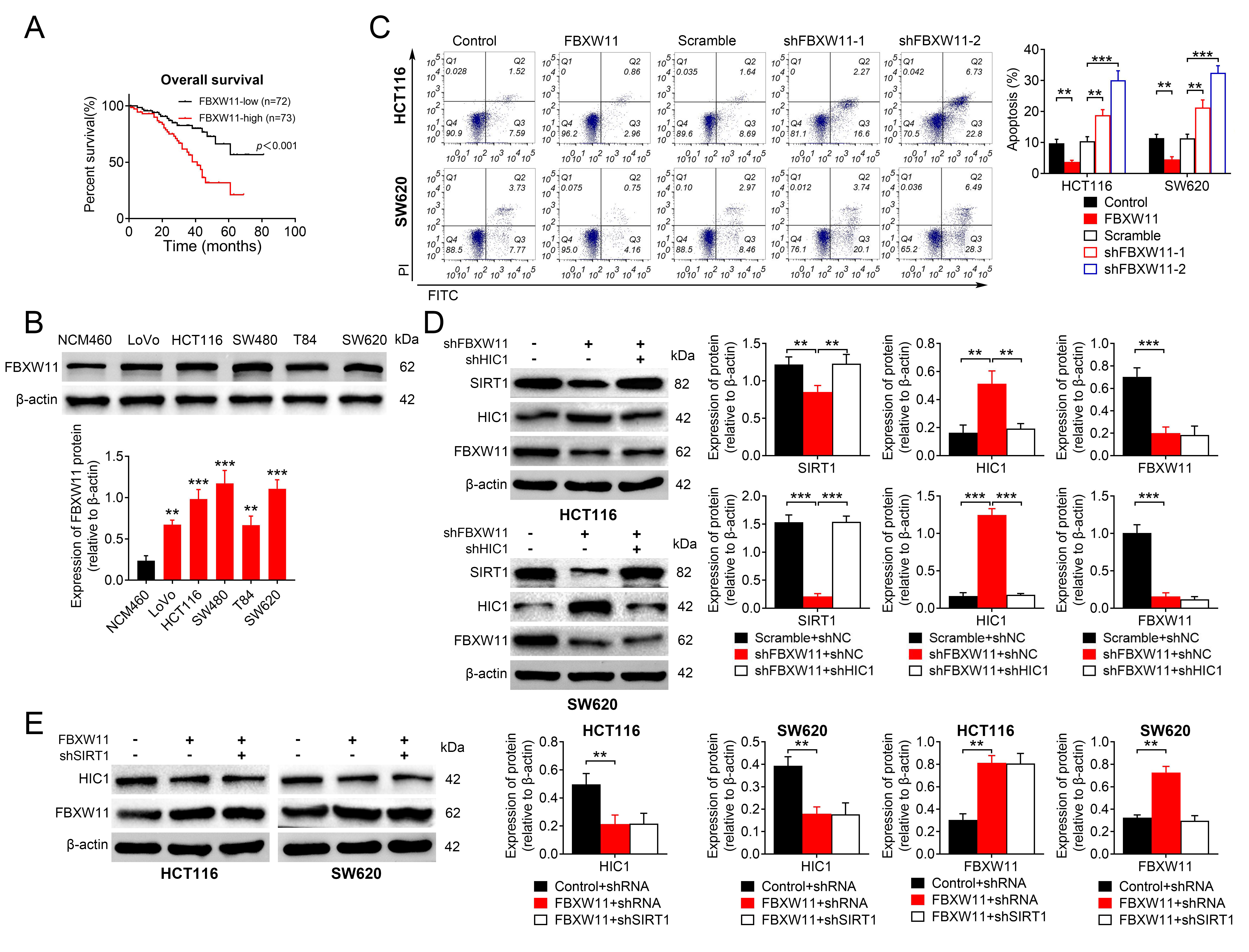


(A) Colorectal tumor tissue and matched adjacent non-tumorous tissues were obtained from 145 patients with CRC. The protein level of FBXW11 in tissue specimens was measure by Western blot. The overall survival of CRC patients with low or high FBXW11 expression was calculated using the Kaplan-Meier method. (B) The expression of FBXW11 in normal human colon mucosal epithelial cell line NCM460 and different CRC cell lines (LoVo, HTC116, SW480, T84, SW620) was measured by Western blot. (C) HCT116 and SW620 cells were transfected with recombinant lentiviral vectors carrying FBXW11 or shRNAs targeting FBXW11 (shFBXW11-1 and shFBXW11-2). The control groups were transfected with corresponding empty control vectors or scrambled shRNA sequence. The apoptotic rate was analyzed by flow cytometry. (D) HCT116 and SW620 cells were co-transfected with shRNAs targeting SIRT1 and/or HIC1 (or scrambled shRNA sequence). The protein expressions of SIRT1, HIC1, and FBXW11 were measured by Western blot. (E) HCT116 and SW620 cells were co-transfected with recombinant lentiviral vectors carrying FBXW11 (or empty control vectors) and shRNA targeting SIRT1 (or scrambled shRNA sequence). The protein expressions of HIC1 and FBXW11 were assessed by Western blot. ANOVA followed by Bonferroni’s post-hoc test was used for statistical comparisons among multiple groups.


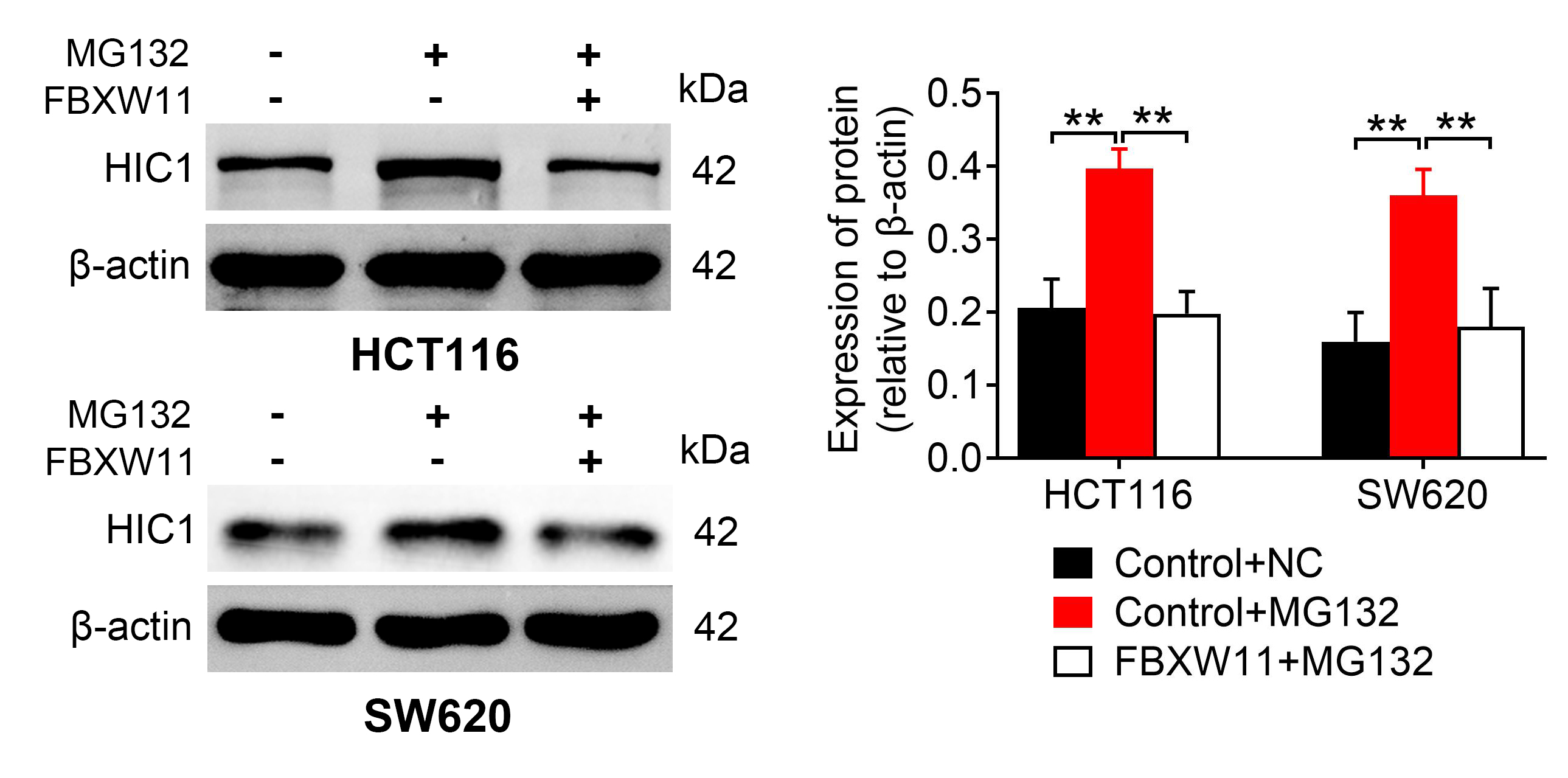


***Supplementary Figure 2.***

HCT116 and SW620 cells were transfected with recombinant lentiviral vectors carrying FBXW11 or empty control vectors. Then, cells were treated with or without 20 μM of MG132 for 3 h. The protein level of HIC1 in different groups of CRC cells was examined by Western blot. ANOVA followed by Bonferroni’s post-hoc test was used for statistical comparisons among multiple groups.
